# Democratising the design and delivery of large-scale randomised, controlled clinical trials in primary care: A personal view

**DOI:** 10.1080/13814788.2023.2293702

**Published:** 2024-01-05

**Authors:** Christopher C. Butler

**Affiliations:** Nuffield Department of Primary Care Health Sciences, Primary Care Clinical Trials Unit, University of Oxford, Oxford, United Kingdom

**Keywords:** General practice/family medicine, general, epidemiology, treatment/intervention research, experimental designs

## Abstract

**Background:**

Rapid identification of effective treatments for use in the community during a pandemic is vital for the well-being of individuals and the sustainability of healthcare systems and society. Furthermore, identifying treatments that do not work reduces research wastage, spares people unnecessary side effects, rationalises the cost of purchasing and stockpiling medication, and reduces inappropriate medication use. Nevertheless, only a small minority of therapeutic trials for SARS-CoV-2 infections have been in primary care: most opened too late, struggled to recruit, and few produced actionable results. Participation in research is often limited by where one lives or receives health care, and trial participants may not represent those for whom the treatments are intended.

**Innovative trials:**

The ALIC4E, PRINCIPLE and the ongoing PANORAMIC trial have randomised over 40,500 people with COVID-19. This personal view describes how these trials have innovated in: **trial design** (by using novel adaptive platform designs); **trial delivery** (by complementing traditional site-based recruitment (‘the patient comes to the research’) with mechanisms to enable sick, infectious people to participate without having to leave home (‘taking research to the people’), and by addressing the ‘inverse research participation law,’ which highlights disproportionate barriers faced by those who have the most to contribute, and benefit from, research, and; in **transforming the evidence base** by evaluating nine medicines to support guidelines and care decisions world-wide for COVID-19 and contribute to antimicrobial stewardship.

**Conclusion:**

The PRINCIPLE and PANORAMIC trials represent models of innovation and inclusivity, and exemplify the potential of primary care to lead the way in addressing pressing global health challenges.

## KEY MESSAGES

Adaptive platform trials can efficiently evaluate several treatments in parallel and sequentially

‘Taking research to people’ can democratise participation by enabling sick, contagious people to contribute from home, country-wide

The PRINCIPLE and PANORAMIC Trials innovated in trial design and delivery to produce evidence on nine treatments for COVID-19 in the community

## Introduction

The World Health Assembly Resolution 75.8 on strengthening clinical trials notes ‘that clinical trials on new health interventions are likely to produce the clearest result when carried out in diverse settings, including all major population groups the intervention is intended to benefit, with a particular focus on under-represented populations’, and calls upon the WHO Director-General to encourage member states to increase the capability for such trials [1].

Communities that bear the greatest burden from infectious diseases and thus stand to benefit most from research findings are typically the most socio-economically deprived and have the least opportunities to participate in trials. During pandemics and epidemics, timely evidence about the effectiveness of early community treatments will have considerable reach and impact. Rapidly identifying treatments that reduce illness burden and limit deterioration helps reduce suffering and supports sustainability of health services and society. Finding out what does *not* work reduces unnecessary exposure to adverse events, reduces waste and exposure to adverse effects, and may enhance antimicrobial stewardship. Findings from efficacy clinical trials, regulatory studies with highly selected participants and hospital-based investigations among the most severely ill, may not always apply to the early treatment of diverse patients in primary care.

This personal viewpoint is based on a plenary address delivered at the World Organisation of Family Doctors (WONCA) Europe conference in Brussels in June 2023. It covers the background, design, implementation, findings, facilitators, and ongoing challenges of conducting large-scale pragmatic platform trials in primary care that may address many of these concerns, and uses the EU Funded ALIC4E, and the UK urgent, national priority PRINCIPLE and PANORAMIC trials as examples. This perspective is rooted in the discipline of primary medical care and draws on experiences of providing personal clinical care and conducting trials in both epidemic and pandemic conditions. The innovations described in trial design and implementation are some of the ways in which primary care can rise to the challenges in the World Health Assembly Resolution on strengthening clinical trials.

## Challenges in infectious disease research in primary care

### The inverse research participation law

In 1971, Julian Tudor Hart, a general practitioner and clinical researcher who practised in Glyncorrwg the Welsh coalfield, articulated the concept of the Inverse Care Law, which states that the “availability of quality medical care tends to vary inversely to the need for it in the population served [2]. It is well-established that individuals living in socially deprived areas experience higher burden of morbidity and mortality [3,4]. For example, those living in the most deprived areas of Wales can expect to live nearly ten years less than those in the least deprived areas [5]. The COVID-19 pandemic further highlighted these health inequalities: As with deprivation generally, deprived regions in South Wales experienced the highest mortality rates from COVID-19.^4^

**Figure 1.**
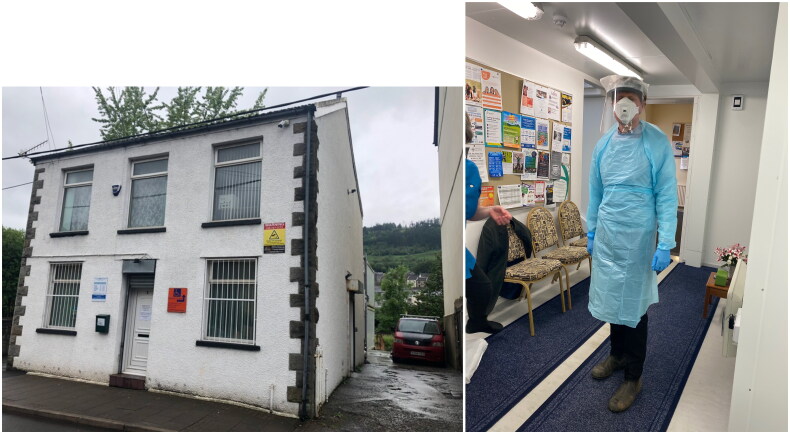
27 March 2020: Chris butler at the Cynon Vale Medical practice.

During the early stages of the SARS-CoV-2 pandemic, while working clinically as a GP at the Cynon Vale Medical Practice, in the Cynon Valley, also in Wales, I managed one of the first patients in the practice with probable COVID-19 (Figure 1). This consultation emphasised not only the lack of evidence-based treatments available for this individual but also an absence of clinical trials available to the patient for addressing this gap. The focus of treatment research, political, and media attention at that time was almost entirely on severe, already hospitalised cases of COVID-19, with limited attention given to community-based therapeutic trials [6]. This led us to formulate the Inverse Research Participation Law, which holds that ‘access to research is often inversely proportional to a participant’s potential contribution, and to where the research findings should be most applicable.’

### ‘Hand me down evidence’ from hospitals research may not apply to primary care

There are critical pathophysiological differences between early and late-stage SARS-CoV-2 infection, requiring distinct treatment approaches [7]. Therefore, evidence from hospital-based studies may not apply to those with early illness [7]. However, given that the vast majority of those unwell with COVID-19 are managed in the community, and only a minority require hospital admission, it is in the community where early, cost-effective, and safe therapeutics could have the greatest gain by preventing progression and transmission, particularly crucial during a pandemic when patient load threatens to overwhelm hospitals.

### Dangers of ‘assumption-based medicine’

Especially in the context of antiviral medication, before widespread use, it is imperative to conduct clinical trials early on to identify those individuals who genuinely benefit from these drugs and those who do not. The parallel pandemic of antimicrobial resistance, partially driven by assumptions as opposed to evidence from clinical trials, serves as a caution. Alexander Fleming famously proclaimed in the 1940s that those who prescribe antibiotics inappropriately must take responsibility for the harm to individuals caused by future, resistant bacterial infections [8]. Antibiotics, including penicillin, were widely prescribed in the community for common infections based on assumptions that they would prevent deterioration and expedite recovery decades before rigorous clinical trials were conducted in this population.

Only in the 1970s did pioneering general practitioners begin conducting randomised controlled trials of antibiotics for common infections that found that most individuals could be safely managed without antibiotic treatment [9–11]. Making the same mistakes and deploying antiviral drugs at scale without proper evidence carries similar risks, including the potential to drive viral mutations with global pandemic potential. The COVID-19 pandemic vividly demonstrates how the world can be upended by a mutation event in a virus, underscoring the importance of evidence-based use of novel antiviral treatment and embedding studies of the effects of therapeutics on the virus in trials.

## Innovative research models

### The 14-country ALIC4E trial of oseltamivir for influenza-like illness

During the ‘swine ‘flu’ (H1N1) pandemic, not a single person in the community was randomised to receive or not receive oseltamivir (Tamiflu), despite it being widely used for this indication. We therefore still do not know whether oseltamaivir helped or harmed people with H1N1 infection, and the drug remains widely contested. Despite recommendations in guidelines, it is hardly used in primary care. Traditional clinical trials have typically been unable to support therapeutic decisions during the same pandemic due to the time and complexity involved in setting up this type of trial [12]. However, the EU-funded PREPARE consortium enabled us to develop a master protocol for a platform trial to test new approaches for conducting community-based trials of antimicrobials for influenza-like illness [13]. An rCt of Clinical and Cost effectiveness in primary CarE (ALIC4E) trial was designed with the flexibility to include new treatments as it progressed and to identify benefits in specific subgroups with combinations of features of interest [14], such as age, co-morbidity and illness duration prior to starting treatment. This 14-country trial, which recruited over 3250 participants, brought us closer to the goal of conducting trials promptly within a pandemic [15].

### Challenges for traditional trials designs under pandemic conditions

Only a small minority of therapeutic trials for SARS-CoV-2 infections have been in primary care: most opened too late, struggled to recruit, and few produced actionable results [12]. Setting up a separate trial for each possible treatment for a novel pandemic infection is likely to be inefficient and so prolong time to results: the analysis of this type of trial only begins once a pre-set, fixed sample size, usually estimated from trials done in another time and place, is reached. This means that recruitment into trials that have larger than anticipated effect sizes may continue beyond the earliest point at which useful findings could have emerged had an interim analysis been conducted before the final sample size was reached. Similarly, an equivocal result could be converted to a clear finding, should recruitment have continued beyond the estimated, fixed sample size. Traditional trials, therefore, do not take data emerging from the trial into account to revise power estimates after randomisation begins. Rather than focusing on which treatment, if any, performs best for a particular condition, such trials only ask one main question: ‘Is this particular intervention more effective than current standard of care or placebo [16]?’ Platform trials, on the other hand, focus more on finding the most effective treatment from several candidates for a particular illness.

### Adaptive platform trials

An adaptive clinical trial allows for aspects of the trial and statistical procedures to be changed after the trial starts without undermining validity or integrity. A platform trial is an adaptive clinical trial in which multiple treatments for the same disease can be tested at the same time and allows for additional treatments to be added while the trial is in progress and for futile interventions to be dropped *via* frequent interim analyses that ensure each drug remains in the trial only until pre-specified thresholds for futility, success or safety concerns are met [13]. A master protocol describes the overall study design, with appendices providing each drug’s details, including additional inclusion criteria and specific monitoring requirements [17].

### The PRINCIPLE trial of repurposed medicines

Through open competition, a national consortium led by the Department of Primary Care Health Sciences at the University of Oxford secured funding to conduct an adaptive platform trial for repurposed drugs to treat COVID-19 in the community. The Platform Randomised trial of treatments in the Community for epidemic and Pandemic iLlnEsses (PRINCIPLE) ­incorporated further innovative features such as response-adaptive randomisation, which allowed the proportions of participants allocated to each intervention to be adjusted based on pre-specified criteria, ensuring that more participants could receive better-performing treatments. Members of the public, expressed surprise that such adaptations were not implemented in all trials [18].

### Open-label trials

The decision not to include a placebo-treated group in PRINCIPLE was driven primarily by the urgency to determine whether prescribing repurposed medicines had an important beneficial impact *compared to not prescribing the drugs*. This contrasts with the question of whether an agent works better than its dummy. Therefore, the appropriate comparator condition was treated without the study drug in question [19,20]. Procuring matched placebo would also have delayed the opening of the trial. Participants receiving a study intervention, regardless of whether it is an active treatment or placebo, are likely to alter their health-seeking behaviour in response to this uncertainty [21], and effect sizes from open trials do not differ meaningfully from placebo-controlled trials [22]. The placebo effect influences cost-effectiveness of healthcare utilisation after treatment - and behaviours are often markedly different in open compared with placebo controlled trials [23]. Thus, an open trial design was also deemed most suitable for a realistic assessment of clinical and cost-effectiveness in practice.

The ALIC4E trial, which employed an open design, yielded treatment effects consistent with the placebo-controlled trials conducted by pharmaceutical companies [15,24,25]. In the event, some of the findings for various drugs from PRINCIPLE’s open trial showed important benefits [26], no benefit [27,28], and suggested harm [29]. As the direction of bias is considered to overestimate benefit in open trials, findings of no benefit, which can have considerable impact on patient safety, costs and antimicrobial stewardship, are likely valid. Open trials do not allow identification of mechanism of action and quantification the magnitude of any placebo effect but can identify or rule out meaningful benefit and impact on outcomes that are unlikely to be influenced by knowledge of treatment allocation. Excluding open trials from systematic reviews and meta-analyses discounts useful safety data and findings on outcome measures unlikely to be affected by a placebo, such as hospital admission for biomarkers. The flexible trial architecture of trials like ALIC4E, PRINCIPLE and PANORAMIC would allow for placebo comparisons.

### Why a pragmatic trial?

Effectiveness trials should include participants similar to those who might be eligible to receive the treatment should it be found to be effective [1], and the study process should reflect real-world care as closely as possible [9,30]. Pragmatism in trial design has been advocated for evaluating the effectiveness of treatments already shown to be efficacious in earlier-phase studies conducted with more selected patient populations, where internal validity is often high but applicability may be questionable [19,30]. Policy makers and primary care clinicians raise questions regarding the generalisability of efficacy trials conducted in controlled settings to real-world patients, who often have multimorbidity and differ by social determinants of health [31].

### Challenges of large-scale community trials

Opening the PRINCIPLE trial presented numerous practical challenges. The UK has over 9000 general practices and not all of them can participate in clinical trial research; very few are affiliated with on-site research pharmacy capability. In contrast, there are fewer than 200 acute hospitals in the UK and all have research-capable pharmacies on site. Despite these challenges, we rapidly opened around 800 practices as trial sites. Clinicians at these sites were required to divide medication into appropriate doses, place them in envelopes, and manually label them for each patient. However, shortly after establishing this process, those with COVID-19 symptoms were advised to use the NHS 111 helpline rather than their general practice. Unfortunately, fragmented nature of the NHS 111 service rendered this recruitment avenue largely unsuccessful.

### Trials that take research to the people (TRTP) vs. ‘de-centralised’ trials

**Figure 2.**
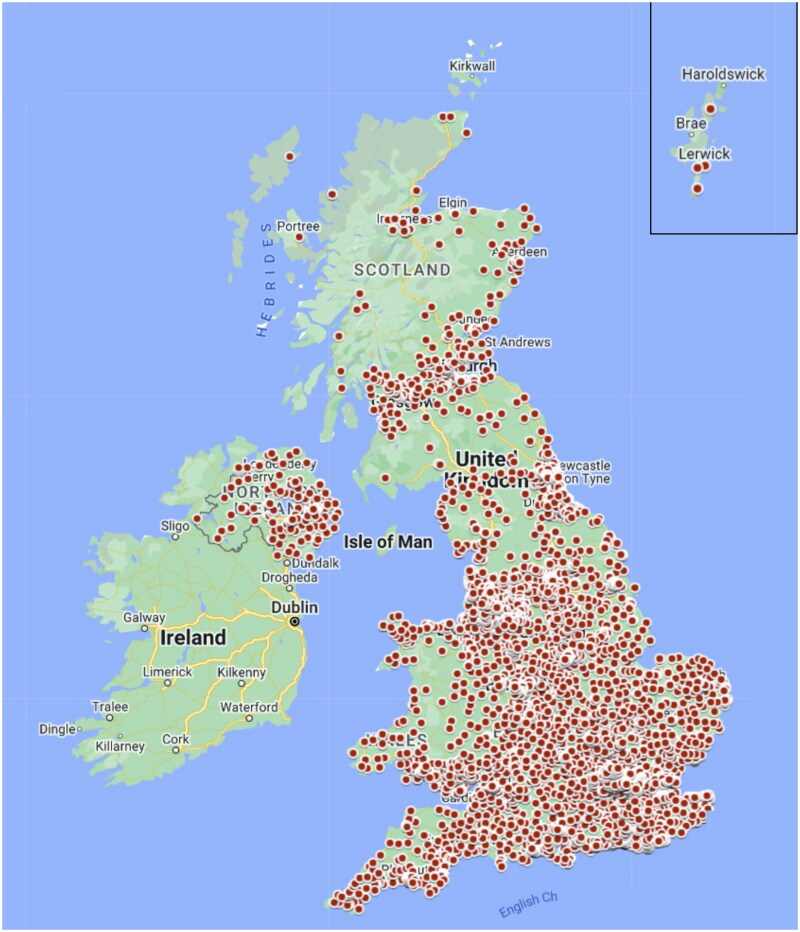
PANORAMIC recruitment from all four UK Devolved Administrations. >120,000 screened 4509 practices referred potential participants to 65 GP practice Hubs accounting for >25% of recruitment: participants came from >6000 GP practices (each red dot represents a GP Practice) Over 3000 randomised to nirmatrelvir/ritonavir vs usual care >25,708 randomised to molnupiravir vs usual care

Traditional models of recruitment into primary care studies rely on recruiting only from those patients who are registered to receive care at GP practices that are set up as research sites. This limits participation according to where one lives and receives health care. To address this, we developed a model to enable participation without leaving home. We obtained permission to inform individuals about the research opportunity *via* email and text messages when they registered for or received COVID-19 test results [32]. Potential participants could then register for the study online and would subsequently be contacted by the study team for eligibility assessment, consent, and, if suitable, enrolment. We obtained permission to use patients’ primary care Summary Care Records as part of the eligibility assessment to ensure safe prescribing in the trial. Medication was couriered to reach participants’ homes usually well within 24 h, regardless of their location in the UK. Recruitment occurred across all four UK Devolved Administrations (England, Wales, Scotland, Northern Ireland), resulting in a truly nationwide sample (Figure 2). This approach has been described as ‘de-centralised,’ when in effect, recruitment becomes largely centralised. Hence, we prefer to call it ‘taking research to the people.’

**Figure 3.**
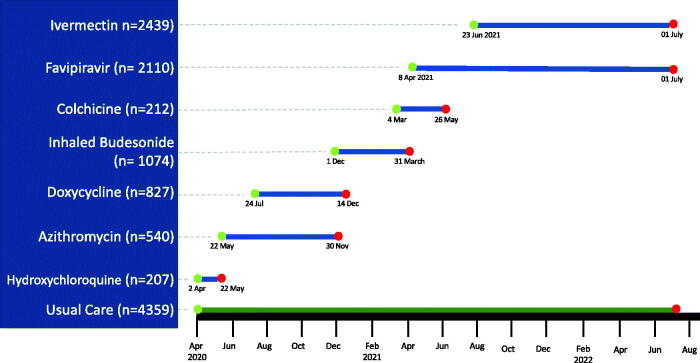
PRINCIPLE trial of repurposed medicines: recruitment timelines.

Participants could nominate a trial partner to respond on their behalf if necessary, providing information through a daily online diary or participating in telephone calls when diary data was not submitted. Individual participant records were linked to national routinely collected data sets to enhance ascertainment of healthcare utilisation and hospital admissions. Digital tools proved critical, contributing to the randomisation and follow up 11,768 people with acute COVID-19 in the PRINCIPLE trial across seven included drugs [32] (Figure 3).

### Key outputs from PRINCIPLE

PRINCIPLE pioneered in demonstrating that commonly used antibiotics, such as doxycycline and azithromycin [27,28], should not be prescribed to treat COVID-19 in the community, in the absence of other indications. During the early stages of the pandemic, these antibiotics were widely used around the world to treat COVID-19 [33], and National Institute for Health and Care Excellence and World Health Organisation guidelines [34,35], as well as many national guidelines were subsequently revised based on our early evidence. These findings impacted antimicrobial stewardship worldwide and contributed to a clinical alert issued by the Chief Medical Officers in the UK, advising primary and secondary care clinicians against using these drugs for COVID-19 treatment. These efforts were recognised through an award by UK Health Security Agencies—the Antibiotic Guardian Awards.

The ColCorona trial demonstrated that the anti-inflammatory drug colchicine [36], when used in the community, was likely to prevent hospital admissions. The efficient PRINCIPLE trial design with its frequent interim analyses allowed us to rapidly establish that colchicine did not improve patient recovery after only 156 participants had been randomised to that study arm [29]. PRINCIPLE found that inhaled budesonide reduced recovery time by approximately three days and there was a high probability that it decreased the likelihood of hospital admission [26]. Papers have been submitted describing the effects of ivermectin and favipiravir treatment for COVID-19 over 28 days, and and on symptoms and function over one year.

### The PANORAMIC trial

Based on evidence from pivotal, company-sponsored trials in unvaccinated populations while the Delta strain was prevalent [37,38], the UK government procured significant quantities of the novel antiviral agents, molnupiravir and subsequently nirmatrelvir- ritonavir. There was controversy about whether to immediately use these drugs as routine treatment for COVID-19 or rather first to conduct a trial to evaluate their effects in a largely vaccinated population when the Omicron variant became dominant. To resolve this dilemma with rigorous prospective evidence and supported by the far-sighted vision of the Chief Medical Officers, the UK commissioned a trial in their intended-use population.

The Platform Adaptive trial of Novel antiviRals for eArly treatment of COVID-19 in the Community (PANORAMIC) enrols individuals between the ages of 18 and 50 with comorbidities, and those aged over 50 regardless of comorbidities, who have been symptomatic for less than five days, tested positive for COVID-19, and exhibited symptoms [39]. The primary outcome is hospital admission, with secondary outcomes reflecting recovery parameters and healthcare utilisation and cost effectiveness, and a virology sub-study enabled by self-swabbing and self-taken blood spot samples.

**Figure 4.**
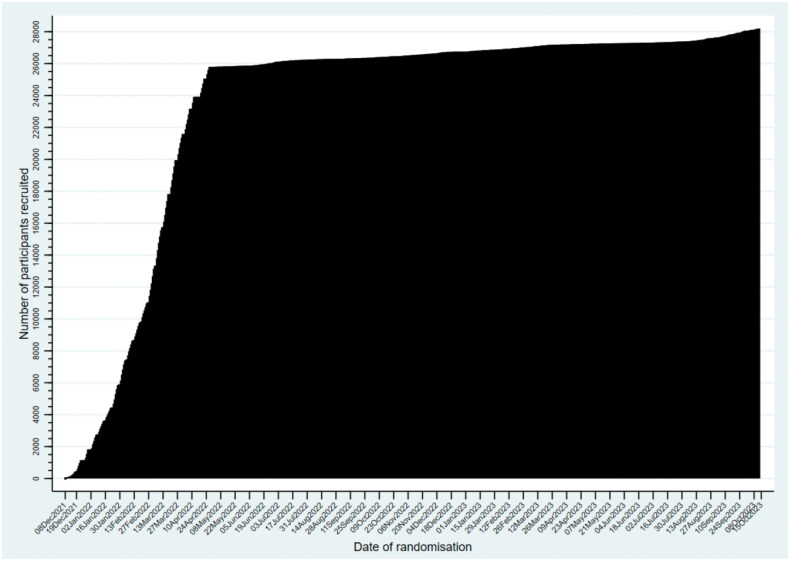
Cumulative recruitment summary into PANORAMIC by 27 November 2023 (*n* = 28,534), trial ongoing.

The trial opened on 8 December 2021, and the molnupiravir arm closed towards the end of April 2022 after randomising 26,411 participants with COVID-19 [40]. On a single day, we randomised over 500 participants (Figure 4). We believe PANORAMIC was the fastest recruiting trial ever of drug treatment in primary care and is the largest therapeutic trial in the community for COVID-19 worldwide. Recruitment to evaluate nirmatrelvir/ritonavir (Paxlovid) is ongoing, and evidence about cost-effectiveness and virological outcomes of molnupiravir are in press. These outputs make key recommendations about the duration of treatment and the price point at which molnupiravir becomes cost effective.

### Public contributors and community engagement

Dedicated public contributors helped guide the trials through membership of the Trial Steering Committees and gave input on the trials’ design and helped develop all patient-facing and trial awareness raising materials and dissemination.

Recognising the importance of addressing health inequalities in research, and specifically to COVID-19, we developed a community outreach program involving faith groups, community organisations, universities, charities, disability groups, and other groups. For instance, student volunteers at the University of Bolton, in an area badly impacted by COVID, received training in the PRINCIPLE and PANORAMIC trials, and wearing hoodies branded with the trial’s logos, engaged in a rolling outreach programme to explain the value of research participation to community and faith groups and in places of high footfall like shopping malls. Our collaboration with the Neasden Temple, the largest Hindu temple outside India, resulted in increased traffic to our website and lasting partnerships and was presented at the United Nations. These and other initiatives contributed to recruiting a sample for the trials that reflected national minority ethnicity in the age groups represented in the trial [41]. The recently launched Centre for Research Equity, through Pharmacy, Communities and Health Care at the University of Oxford, builds on such engagement.

### PANORAMIC’s main findings thus far

The primary outcome measure of hospital admission was captured for over 97%, with over 90% completing daily diary data. In the virology sub-study, over 80% provided self-collected blood samples and swabs [40]. The trial population was almost entirely vaccinated, and participants were randomised within a median of two days from symptom onset and received medication mostly within five days. Over 90% of participants completed the molnupiravir course. We found molnupiravir used early in the community by vaccinated individuals during the Omicron variant’s circulation did not reduce already low hospital admission rates. However, we observed a substantial benefit in terms of early recovery, reduced healthcare utilisation, and decreased viral detection and load. The drug was well-tolerated and deemed safe [40].

## Facilitators and challenges

### Key Facilitators

The experience of conducting a trial of a therapeutic for influenza-like illness during ‘peacetime’ and novel methods such as the adaptive platform design was central to rapid initiation of our COVID-19 trials [15]. Rapid, peer-reviewed funding processes allowed experienced teams to initiate trials promptly. Within weeks, several hundred trial sites were established: The Urgent Public Health Panel prioritised resources for national platform trials, with PRINCIPLE and PANORAMIC receiving priority support for primary care recruitment from The National Institute of Health Research (NIHR) Clinical Research Network (CRN) infrastructure, Health and Care Research Wales, NHS Research Scotland and the Health and Social Care Board in Northern Ireland.

Working with the NIHR, and the CRN meant that the standing clinical research infrastructure in NHS primary care was stood up to support the studies. The UK is unique in having this infrastructure exclusively devoted to efficiently delivering studies on its portfolio, predominantly in the NHS. Collaborative working with the primary care team in the NIHR was a key factor to the efficient and at-scale delivery, mainly when ‘hub and spoke‘models of recruitment were used: Over 4500 ‘spoke’ practices could refer potentially eligible patients to 65 research ‘hub’ practices for completing consenting and study procedures. The CRN provided research staff at many of the English hub sites to facilitate recruitment, promoted the study across the network, liaised with NHS England and UK government and monitored recruitment through a dedicated app.

The COVID-19 Therapeutics Advisory Panel independently assessed potential interventions to guide selection for the trials. Early engagement with regulatory bodies, such as the Health Research Authority and the Medicines and Healthcare Products Regulatory Authority, facilitated swift trial opening and amendment approvals. Digital tools, data linkage, remote consent, and direct medication delivery to participants’ homes, the facility to nominate a trial partner to provide data when the trial participant could not do so, allowed us to complement recruitment through General Practices complimented by decentralised trial capability.

### Ongoing challenges

Funding models that favour medicine-specific trials, rather than maintaining trial-ready infrastructure to answer questions about a range of treatment options for an illness, are generally ill-suited for pandemic and epidemic therapeutic trials in primary care. To initiate trials rapidly, ethical and regulatory approval needs to be expedited; contracts, permissions, and protocols should already be in place; and sites experienced in trial delivery must be available. This requires a robust standing infrastructure that includes adequate pharmacy support. Contracts and regulatory review should be proportionate to risk, with an understanding by regulators that processes appropriate for earlier phase studies in clinical research facilities might not apply to pragmatic community-based research of low-risk interventions.

Regulations often do not adequately support data acquisition for patients who have already given their consent for trialists to use their data. Improved communication on Bayesian statistics and probability is necessary to include evidence from such trials into clinical guidelines and facilitate widespread uptake of findings.

PANORAMIC and PRINCIPLE may give the impression of being just two trials, but they represent nine treatment evaluations, and each intervention requires all the usual trial processes. For example, while we were introducing a new drug with all that entails, we might have been analysing the results for another drug, responding to reviewer’s comments for another output, working on permissions for and implementing innovations about data access and capture, and contributing to regular meetings of bodies such as the EU Covid-19 Trials Coordination Board, the Covid-19 Therapeutics Advisory Panel, the Urgent Public Health Panel, the Antiviral Task Force, the World Health Organisation Covid Outpatients Working Group, and the NIHR Clinical Research Network. Our recruitment team worked six days each week and our safety team covered every day of the year. Obtaining exemptions and permissions required many lengthy justifications and meetings with numerous bodies and individuals: it was not unusual for our first meeting to begin at 7am and to progress wall-to-wall until 9 pm. Every day was a working day in the first years of the pandemic. We lost several key research professionals to industry mid-pandemic, with offers of more money and better terms of service. Academic Trials Units may be best equipped to design and implement policy-relevant trials but this depends on competitive contracts and not short term that, lurching form study to study. While efficiencies come from adaptive platform trials, funding should recognise the multiplicity of tasks being attended to simultaneously by the multidisciplinary team.

Academic ambition and rivalry led to research wastage. Rather than ‘do it once and do it right’ internationally, we find 280 trials hydroxychloroquine registered on Clinicaltriasl.gov! It was surprising to learn how many expert clinical trialists of therapeutics for infectious disease in the community there suddenly were, and how many people had unique and passionate insights into agents that would cure COVID-19! Repeated Freedom of Information requests, usually about politically charged interventions slowed us down further.

Nationalism and lack of harmonised regulations and disproportionate processes undoubtedly limited collaboration across borders, with each country generally resorting to setting up its own trials. We worked hard with colleagues in several countries to extend the trials internationally but largely failed due to national funding limitations and regulatory and legal hurdles. Internationally conducted trials are likely to yield results faster and overcome challenges associated with competition for trial sites and concerns about publication credit. A trial platform should be viewed as a common good, allowing academic and commercial groups to propose interventions for independent evaluation.

## Conclusion

Adaptive platform trials, which can evaluate multiple treatments in parallel and in series, are particularly suited for evaluating therapeutics in pandemic and epidemic conditions but are potentially applicable to a wide range of conditions in primary care, including non-communicable illness. By combining traditional community-based sites in general practices with taking research directly into people’s homes, we can address the inverse research participation law. Rapid initiation of trials evaluating novel antimicrobial drugs in the intended-use population is crucial not only for pandemic preparedness but also for preventing future catastrophes arising from the assumption-based use of antimicrobials. Such an approach must be evidence-based, derived from prospective randomised controlled trials that are: prioritised, coordinated, mandated, resourced, embedded in primary care, sustained, simplified, and democratised. The PRINCIPLE and PANORAMIC trials represent models of innovation and inclusivity and exemplify the potential of primary care to lead the way in addressing pressing global health challenges through ‘well designed and well implemented clinical trials that include all the major population groups the intervention is intended to help’ that the World Health Assembly calls for [1].
